# Medical and Dental Students’ Perceptions and Attitudes towards Interprofessional Clinical Education

**DOI:** 10.15694/mep.2018.0000249.1

**Published:** 2018-11-08

**Authors:** Sanghamitra M. Misra, Shawn Adibi, Margo Melchor, Liang Zhu

**Affiliations:** 1 Baylor College of Medicine; 2 The University of Texas School of Dentistry at Houston; 3 The University of Texas Health Science Center at Houston

**Keywords:** Dental education, Medical education, Interprofessional education, IPE, clinical IPE

## Abstract

This article was migrated. The article was marked as recommended.

**Purpose**: Interest and expansion of interprofessional education (IPE) has increased tremendously over the last decade due to need and regulatory requirements.

**Methods**: Third-year medical students and third and fourth-year dental students participated in a combined IPE experience at a dental assessment clinic. All participating students completed the Readiness for Interprofessional Learning Scale (RIPLS) Questionnaire before the session, and medical students completed an evaluation after the session.

**Results**: All students agreed that IPE is important and needed for the development of skills, and they see benefit to shared learning. The only RIPLS question in which students did not agree was “Clinical problem solving can only be learnt effectively with students/ professionals from own school/organization.” The dental students agreed with this more than medical students (p=0.04). Age of medical and dental students was related with outcomes of RIPLS questions 2, 7, 14, and 16 (p=0.03, 0.02, 0.03, and 0.02, respectively). Older participants from both schools tended not to agree with statements related to importance of working together benefiting patients, improving working relationships, welcoming the opportunity to work on small group projects with other health/social care students, and whether shared learning/practice will help clarify the nature of patients’ problems. Medical student evaluations of the dental IPE experience were very positive, and mild changes in the curriculum improved medical student perception of the experience from year 1 to 2.

**Conclusion**: Guidelines and standardized curricula could help medical and dental school faculty create clinically appropriate and effective IPE interactions for learners of all ages.

## Introduction

Interprofessional education (IPE) has been included in health professional curricula since being introduced in the 1970s, but interest and expansion of IPE has increased tremendously over the last decade due to evident need as well as regulatory requirements from Liaison Committee on Medical Education (LCME) for medical students and Commission on Dental Accreditation (CODA) for dental students. Through IPE, students are trained to understand roles and responsibilities of other professions and to work collaboratively with other healthcare professions. The expected direct effect of IPE is to improve overall patient health. Pediatric IPE experiences including dental, nursing, and osteopathic medical students have been shown to create statistically significant improvement in the overall knowledge of children’s oral health topics, confidence in their ability to provide oral health services, and clinical practice. ( Cooper et al., 2017) IPE including nurse practitioner/ midwifery, dental, and medical students has also shown to be effective by using clinical simulation and case studies. ( Haber et al., 2017) Even IPE with dental hygiene students teaching medical and dental students has been effective. ( Otsuka et al., 2016) Despite the potential positive effects of IPE, challenges exist in creating new health care curricula. Interestingly, when looking at attitudes about IPE, one published evaluation of an introductory IPE course for healthcare students using the Readiness for Interprofessional Learning Scale (RIPLS) and the Interdisciplinary Education Perception Scale (IEPS), the IPE did not positively affect student attitudes towards other professions, strengthen professional identity, nor improve readiness for interprofessional learning. The results of the study supported Kegan’s theory of professional identity formation, which describes a process by which individuals move through stages of identity in an effort to construct an understanding of what it means to belong to their professions. Students in the study were found to be in the first stage of professional identity formation, which is characterized by self-orientation and lacking a broader view of professionalism that includes roles outside of their profession. ( Stull and Blue, 2016) Although this was a relatively negative study, it is important to note that IPE is still useful in helping students learn about their own role relative to other healthcare professional roles. For medical education faculty, it is vital to create experiences throughout the various years of medical education and to set realistic expectations of all curricula.

In an effort to improve patient health for their patients, Baylor College of Medicine (BCM) and UT Health School of Dentistry (UTSD) collaborated to create a combined clinical experience for their respective medical and dental students. The IPE experience was the first and only IPE for both the schools combining medical and dental education. The purpose of this study was to evaluate all the students’ attitudes and perceptions about IPE and to explore medicals student perceptions about this IPE experience.

## Methods

This study was reviewed and approved by the Institutional Review Board (IRB) of UTHealth (Protocol Number HSC-DB-17-0006) and reciprocated by the IRB of Baylor College of Medicine (Protocol Number H-40661). This project was partly supported by funds from the National Institutes of Health, National Heart, Lung, and Blood Institute grant number R25 HL 108183-01.

Third-year medical students and third and fourth year dental students were chosen to participate in a combined IPE experience at the UTSD Assessment Clinic. The IPE occurred on most Thursdays during the school year. Medical students were required to participate in the IPE as part of their enrollment in a course named Longitudinal Ambulatory Care Experience (LACE). LACE is a required third-year course at BCM which seeks to raise awareness and promote medical student competency in managing the medical, social, spiritual, and cultural needs of patients by combining outpatient preceptorship experiences and community experiences such as protective services, palliative care, hospice, and clinical home visits. Dental students were required to participate in the IPE as part of their enrollment in either the clinical assessment rotation named CLIN 3017 (Third-year Assessment, Diagnosis and Treatment Planning Clinical Course) or CLIN 4012 (Fourth-year Assessment, Diagnosis and Treatment Planning Clinical Course).

All participating medical and dental students were asked to complete the Readiness for Interprofessional Learning Scale (RIPLS) Questionnaire before the IPE clinic session. However, completion of the survey was voluntary. The clinical assistant in the UTSD Assessment Clinic collected the questionnaires. During the IPE session, medical students were paired with dental students and all learners were supervised by a UTSD dental faculty member. Each week, medical students were paired with dental students and each medical student/dental student group assessed one new patient. Ideally, there was a ratio of one medical student to one dental student. However, there were often two dental students per one medical student. The goals for this experience were for both groups of students to better communicate one’s roles and responsibilities clearly to patient, family community member and other professional as well as recognize one’s limitations in skills, knowledge and abilities. To meet this goal, the medical student objectives were to learn (1) how dentists conduct an oral health assessment, (2) the aspects of a comprehensive dental exam, (3) how to order proper dental diagnostics and referrals, and (4) how to interact/work with other healthcare professionals. Dental students learning objectives were to learn (1) how to take a medical comprehensive history, (2) how to interpret normal and abnormal findings on the medical history, and (3) how to interact/work with other healthcare professionals. During the patient appointment, the medical and dental students worked together to review the patient’s chief complaint and medical history. In addition, need for radiographic images were determined based upon the patient’s dental condition. A UTSD dental faculty member concluded the patient encounter by reviewing the history, conducting the physical exam and concluding the visit. After the clinical portion of the rotation, a UTSD dental and/or dental hygiene faculty member provided an interactive lecture session on a topic of their expertise that was relevant to medical students. Please see Table 1 for the Medical/Dental IPE curriculum design. Over a two-year period of offering this curriculum, all the medical students were required to complete an evaluation of the IPE experience to improve the quality of the session. The evaluations from the first year of the curriculum were used to improve the session for the second year.

**Table 1.** The Medical/Dental IPE curriculum design

Task	Person in charge	Notes
Created partnership between medical and dental school	Course director at the medical and Clinical lead at dental school	Ours was inter-institutional so the IPE experience required significant planning
Created memorandum of understanding (MOU) and education affiliation agreement between medical and dental school	Course director at the medical and Clinical Lead at dental school	Agreements took 2 years to complete.
Determined optimal learning activity for the 2 groups (clinical experience in school of dentistry dental assessment clinic)	Course director at the medical school and Clinical Lead at dental school	Interactive learning is often well received so opted for clinical experience
Scheduled weekly sessions to accommodate various medical and dental school schedules	Clinical Lead at dental school	The medical student time was fixed, so dental clinic was chosen accordingly.
Created curriculum with details of goal, objectives, agenda, number of learners, and number of faculty	Course director at the medical and Clinical Lead at dental school	Created our own curriculum as no available curricula suited our needs
Invited dental school faculty to give a lecture at the end of each clinical IPE experience	Clinical Lead at dental school	Rotated different dental subspecialties
Created IPE Experience Schedule	Course director at the medical school and Clinical Lead at dental school	-4 Medical students reported to dental school -Dental faculty member introduced everyone and paired each medical student with one dental student (dyads) -Dental faculty member reviewed objectives and emphasized importance of IPE/ student interactions -Each dyad assessed one dental patient -Each dyad reported findings to an available Dental faculty member and watched faculty member complete history, physical, and assessment -Medical and dental students were given time to ask questions of each other -Dental school faculty member provided lecture relevant to medical students

## Results/Analysis

STATISTICAL ANALYSIS: Summary statistics were provided for the demographic variables of the participants and their distributions were compared between medical and dental school students by Chi-square test or Wilcoxon rank sum test.

For the medical and dental student RIPLS questionnaire, frequency and relative frequency of answering agree (“agree” or “strongly agree”) were provided. For each question, the frequencies of answering agree were compared between the medical and dental students by Chi-square test or Fisher’s exact test. Finally, the association between the outcome of each question and other factors including gender, previous experience, and age were evaluated by Chi-square test, Fisher’s exact test, or Wilcoxon rank sum test. All analysis were performed in SAS 9.4 (Cary, NC).

RESULTS: In total during the RIPLS study period, 22 medical students and 53 dental students participated in this IPE experience. Of those students, 21 medical students and 30 dental students completed the RIPLS (68% overall response rate). Table 2 delineates demographics of responders.

**Table 2.** Demographics for RIPLS Study

Variable		Medical Students (n=21)	Dental Students (n=30)	P value
**Gender**	Male	16 (76.19%)	15 (50%)	0.06
	Female	5 (23.81%)	15 (50%)	
**Previous IPE Experience**	Yes	9 (42.86%)	7 (24.14%)	0.16
	No	12 (57.14%)	22 (75.86%)	
**Mean Age**		24.95 years (SD 1.2)	26.77 years (SD 2.88)	0.01

The medical and dental students were similar in gender and any previous IPE experience, but dental students were older than medical students (24.95 vs. 26.77, p=0.01). Medical students were all third-year students while the dental students were both third and fourth year students. So, this may be one reason for the noted difference. The medical and dental students’ responses to the RIPLS questionnaire were very consistent. Both medical and dental students agreed that IPE is important and needed for the development of their skills, and they see benefit to shared learning. The only RIPLS question in which the medical student and dental students did not agree was #12-“Clinical problem solving can only be learnt effectively with students/ professionals from my own school / organization.” The dental students agreed with this more than the medical students (p=0.04). Since the field of dentistry is relatively narrow in its focus compared to medicine, it is possible that the dental students feel that their own faculty are better able to teach them about dental clinical problem solving. On the other hand, medical students and physicians are often asked questions from patients related to their mouth and teeth. So, it is possible that medical students feel that they can learn clinical problem solving skills from dental students and dentists since the ideas are relevant to their own practice.

The association between the outcome of each question and other factors including gender, previous experience, and age were evaluated by Chi-square test, Fisher’s exact test, or Wilcoxon rank sum test. All analysis were performed in SAS 9.4 (Cary, NC). Gender and previous IPE experience were not related with the outcomes of any question. Age of medical and dental students, as a continuous variable, was related with the outcomes of question 2, 7, 14, and 16 (p=0.03, 0.02, 0.03, and 0.02, respectively). Older participants from both schools tended not to agree with the statements in these questions. See Table 3 for RIPLS responses.

**Table 3.** Frequency and relative frequency of answering “agree” or “strongly agree” in all participants and in medical and dental students, respectively

		Medical Students N (%)	Dental Students N (%)
**1**	Learning with other students/ professionals will make me a more effective member of a health and social care team	21 (100%)	29 (96.7%)
**2**	Patients would ultimately benefit if health and social care students/ professionals worked together	21 (100%)	28 (93.3%)
**3**	Shared learning with other health and social care students/ professionals will increase my ability to understand clinical problems	21 (100%)	28 (93.3%)
**4**	Communication skills should be learned with other health and social care students/ professionals	20 (95.2%)	29 (96.7%)
**5**	Team-working skills are vital for all health and social care students/ professionals to learn	21 (100%)	30 (100%)
**6**	Shared learning will help me to understand my own professional limitations	21 (100%)	26 (86.7%)
**7**	Learning between health and social care students before qualification and for professionals after qualification would improve working relationships after qualification/ collaborative practice	21 (100%)	26 (86.7%)
**8**	Shared learning will help me think positively about other health and social care professionals	20 (95.2%)	25 (83.3%)
**9**	For small-group learning to work, students/professionals need to respect and trust each other	21 (100%)	30 (100%)
**10**	I don’t want to waste time learning with other health and social care students/ professionals	1 (4.8%)	3 (10%)
**11**	It is not necessary for undergraduate/postgraduate health and social care students / professionals to learn together	0 (0%)	3 (10%)
**12**	Clinical problem solving can only be learnt effectively with students/ professionals from my own school / organization	0 (0%) *	6 (20%) *
**13**	Shared learning with other health and social care professionals will help me to communicate better with patients and other professionals	21 (100%)	29 (96.7%)
**14**	I would welcome the opportunity to work on small group projects with other health and social care students/ professionals	19 (90.5%)	23 (76.7%)
**15**	I would welcome the opportunity to share some generic lectures, tutorials, or workshops with other health and social care students/ professionals	18 (85.7%)	27 (90%)
**16**	Shared learning and practice will help me clarify the nature of patients’ or clients’ problems	21 (100%)	26 (86.7%)
**17**	Shared learning before and after qualification will help me become a better team worker	20 (95.2%)	28 (93.3%)
**18**	I am not sure what my professional role will be/is	0 (0%)	2 (6.7%)
**19**	I have to acquire much more knowledge and skill than other students/ professionals in my own faculty/ organization	7 (33.3%)	10 (33.3%)

* indicates that there is a significant difference between medical and dental students.

Over a 2 year time period, 68 third-year medical students (19 in the first year and 49 in the second year) participated in this medical/dental IPE curriculum. The RIPLS study period was only 12 of 20 weeks in the second year of the curriculum offering. Overall, the medical students felt that the experience was well received, informative, and helped them understand the role of a different field of medicine. In the first year, the students did not respond as positively that the IPE experience helped them improve effective communication with other healthcare professionals. Between years 1 and 2 of the curriculum, the course director and clinical lead of the dental clinic reviewed the evaluations and made changes to improve certain aspects of the curriculum such as improving the introduction at the beginning of the session so that all students understood their roles and responsibilities. In the second year of the curriculum, during an introductory session for the course, the medical school course director also emphasized the importance of each student sharing and answering questions during IPE to foster better communication and collaboration. With small tweaks in the curriculum, in the second year, overall more students felt that their communication was improved with this IPE experience. Table 4 reports quantitative responses about the experience comparing years 1 and 2. Tables 5 and 6 lists verbatim comments about the learning experience and areas of improvement, respectively.

**Table 4.** Dental IPE Medical Student Quantitative Evaluations

	n	Mean	Scale	Std
First Year Curriculum Evaluation Data				
The facilitator(s) were prepared for LACE students on the day I attended.	19	5.05	1 to 6	1.43
I felt welcome at this community site.	19	5.68	1 to 6	0.48
This community site helped me to gain knowledge about common and/or chronic medical disorders treated in an ambulatory setting.	19	5.26	1 to 6	0.99
This community site helped me to practice patient care skills.	19	4.84	1 to 6	1.50
This community site helped me to improve effective communication with patients, physicians, nurses, social workers and/or other health care professionals.	19	4.89	1 to 6	1.41
This community site helped me to increase my awareness about the roles of non-physician health care providers and the delivery of health care services in the community.	19	5.53	1 to 6	0.61
This community site helped me to increase my awareness about the social, financial, and/or compassionate challenges of health care.	19	5.11	1 to 6	1.20
**Second Year Curriculum Evaluation Data**				
I felt welcome at this community site.	49	5.67	1 to 6	0.63
Had you ever visited this community site before LACE as part of your medical education?	49	1.98	1 (yes) 2 (no)	0.14
This community site helped me to improve effective communication (e.g. with patients, physicians, nurses, social workers and/or other health care professionals).	49	5.43	1 to 6	1.10
This community site helped me to increase my awareness about the roles of non-physician health care providers.	49	5.57	1 to 6	0.94
This community site helped me to increase my awareness about the delivery of health care services in the community.	49	5.57	1 to 6	0.89
This community site helped me to increase my awareness about the social, financial, and/or compassionate challenges of health care.	49	5.53	1 to 6	0.82
This community site helped me to practice inter-professional education in clinical settings with non-physician health care providers.	49	5.57	1 to 6	0.87
Overall, this was an excellent community site experience.	49	5.49	1 to 6	0.98

**Table 5.** Strengths of the IPE Experience (Selected Verbatim Medical Student Comments)

Please describe the strengths of this learning experience.
Very good coverage of all the services dentists provide and how they collaborate and work with physicians.
Extensive interaction with non-physician health personnel, especially dental students. Information about their different clinics and the services offered was very interesting and relevant for patients that we see during clinical rotations.
It was helpful to learn more about dentistry. It’s interesting how in medicine, we take care of most of the body, but don’t learn too much about the teeth and oral care. Though I understand the need for specialization, it’s good to learn more and be more knowledgeable about an important area of the body.
Wonderful! I finally learned what our dental colleagues do! This should almost be mandatory- oral health is so important and represents a gap in our current curriculum. Our hosts at UTHSC were fantastic and made me feel very welcome as part of their team. Great experience; would strongly recommend.
Great to learn about this side of healthcare. I had never interacted with a dentist in an inter-professional setting, and I thoroughly enjoyed learning about dentistry and working with dental students.

**Table 6.** Areas of Improvement (Selected Verbatim Medical Student Comments)

Please describe areas of improvement for this learning experience.
The only portion I would change is the lecture component. I’m not sure lecture is a great format for that setting. Our exposure is so limited that almost anything showing us actual dental things would be helpful while a lecture didn’t seem like a great use of time. (That said, it was a good lecture and I appreciated the efforts that were involved.)
I think this would be a more beneficial learning experience if the lectures focused on basic dentistry that medical providers must know; such as when to send someone to a dentist or how to deal with dental emergencies.
I wish there had been more instruction on when to refer to a dentist for problems. I also feel like watching the evaluations for more than an hour becomes redundant.. Also, I felt that the 30-minute presentation at the end on domestic violence had nothing to do with the rest of the visit, and would have appreciated being able to instead talk to a dentist more in-depth about how their healthcare system worked.

## Discussion

Overall, the medical dental IPE was a success at our institutions. When examining the RIPLS responses, the students generally agreed that IPE is important and needed for the development of their skills, and they see benefit to shared learning. The only RIPLS question in which the medical student and dental students did not agree was #12-“Clinical problem solving can only be learnt effectively with students/ professionals from my own school / organization.” The dental students agreed with this more than the medical students (p=0.04). It is possible that dental students find their own dental faculty more knowledgeable and able to help them learn about unique dental clinical problem solving. These types of nuances are important to note as IPE curricula are developed in medical and dental schools. Likely for each profession, IPE is more appropriate for certain core competencies but less appropriate for others. Older participants from both schools tended not to agree with the statements in four questions- numbers 2, 7, 14 and 16. Interestingly, all 4 of these questions are related to health care professionals from different fields working together to learn from each other and improve patient care. This may be explained by the fact that older patients have various life experiences, and they may not feel that IPE will enhance their already broader knowledge base. Although these types of responses may be concerning for IPE-minded faculty, increasing IPE in medical and dental curricula may change students’ minds about the benefits of IPE and interprofessional collaboration.

The medical student quantitative evaluations of our IPE curriculum are very encouraging. Students were able to improve their communication skills and learn from non-physician health professionals on how they deliver care to their patients. The students overall felt welcome at the dental clinic site and enjoyed the experience. The qualitative comments about the IPE experience were very positive. The students clearly appreciated the opportunity to work in a different type of clinic setting and learn about a field of medicine that is so closely related to their own practice but yet so different. There were very few comments made about areas of improvement, but the available comments were constructive. After the first year of the curriculum, dental faculty modified the session to improve the interactions between all students and by providing more relevant lecture topics for medical students. One limitation of our study is that we did not collect evaluations of the IPE curriculum from the dental students. However, from the dental student RIPLS responses, it is clear that they in general value IPE experiences within their dental education.

## Conclusion

Medical and dental students are consistently interested in the benefits of interprofessional education and are willing to engage in IPE experiences. Medical students working alongside dental students in a dental assessment clinic is a beneficial setting especially for medical students to learn about the practice of dentistry. Dental students can teach medical students about their history and physical. At the same time, medical students can answer dental student questions related to general medical patient care. Since medical and dental IPE curricula are relatively new and evolving quickly, further research and guidelines are needed to help medical and dental school faculty create the most clinically appropriate and effective IPE interactions for learners of all ages.

## Take Home Messages

• Medical-dental IPE needs to be tailored to the competencies that can be taught successfully in a group IPE setting.
• Older students may have different needs than younger students in IPE.
• Evaluations from IPE curricula from students can improve local programs.

## Notes On Contributors

Dr. Sanghamitra M. Misra is Associate Professor of Pediatrics at Baylor College of Medicine (BCM), former course director of the BCM course LACE, Medical Director of the Texas Children’s Mobile Clinic Program, and Medical Director of the Texas Children’s Hospital Integrative Medicine Program. Her research interests include interprofessional education, wellness/professionalism, and integrative medicine.

Dr. Shawn Adibi is an Associate Professor of The University of Texas School of Dentistry at Houston. He is an academic fellow of American Academy of Oral Medicine (AAOM). His clinical researches and activities are focused on temporomandibular disorders and oral medicine as it relates with integration of medicine and dentistry.

Dr. Margo Y. Melchor is the Director of Community Outreach and an Associate Professor of The University of Texas School of Dentistry at Houston. She also holds a teaching position in the Department of Periodontics and Dental Hygiene, as well as with the pre-doctoral program in community service learning and geriatric education.

Dr. Liang Zhu is an Associate Professor the University of Texas Health Science Center at Houston (UTHealth) McGovern Medical School. Dr. Zhu’s current research focuses on longitudinal data analysis, survival analysis, semiparametric methods, as well as applications in epidemiology and clinical trials.

## References

[ref1] Cooper D Kim J Duderstadt K Stewart R (2017) Interprofessional Oral Health Education Improves Knowledge, Confidence, and Practice for Pediatric Healthcare Providers. Front Public Health. 14(5), pp.209. 10.3389/fpubh.2017.00209 PMC5557781 28856134

[ref2] Haber J Hartnett E Allen K Crowe R (2017) The Impact of Oral-Systemic Health on Advancing Interprofessional Education Outcomes. J Dent Educ. 81(2), pp.140–148. 28148604

[ref3] Otsuka H Kondo K Ohara Y Yasuda M (2016) An Inter- and Interprofessional Education Program in Which Dental Hygiene Students Instruct Medical and Dental Students. J Dent Educ. 80(9), pp.1062–1070. 27587573

[ref4] Stull CL and Blue CM . (2016) Examining the influence of professional identity formation on the attitudes of students towards interprofessional collaboration. J Interprof Care. 30(1), pp.90–96. 10.3109/13561820.2015.1066318 26833108

