# Supplementary material for: Medical and Dental Students’ Perceptions and Attitudes towards Interprofessional Clinical Education
Source: MedEdPublish (2016). 2018 Nov 8;7:249. [Version 1] doi: 10.15694/mep.2018.0000249.1 (PMC10711984; doi:10.15694/mep.2018.0000249.1)
Supplement: Peer review history

# Reviewer report: Reviewer response for version 1

**DOI:** 10.21956/mep.19712.r29498

**Published:** 2018-11-15

**Authors:** Tarun Sen Gupta

**Affiliations:** 1 James Cook University

This review has been migrated. The reviewer awarded 4 stars out of 5 The authors are to be congratulated on this interesting approach which addresses (in my mind, at least) an under-explored area in most medical and dental curricula, namely an understanding for each of what the other profession involves. In my experience medical students can graduate with little more than a lay understanding of dentistry, yet dental problems, including emergencies are common in primary care.This novel approach uses peer:peer teaching, which is efficient as it does not rely on additional staff resources, as well as promoting connect ions between individual students and developing their teaching skills. The authors may wish to develop this point a little more, or explore it in subsequent evaluations. Other aspects to consider in the future could include longer-term evaluations and higher levels of Kirkpatrick’s hierarchy i.e. exploring ‘learning’ or even ‘behaviours’ rather than ‘reactions’.I was not sure from the description how many visits each student made to the clinic (apologies if I missed this detail) but I think it would be worth including / discussing). The post-visit lecture is also an interesting idea, and invites further discussion: would a briefing lecture pre-visit be worthwhile? Would some sort of standard recording about common problems followed by Q & A be helpful?While this intervention is set in the USA context it is worthy of dissemination and consideration in other settings as it is feasible and seems to meet an important need, addressing learning gaps of students in both disciplines. Perhaps another form of symbiotic education?

Reviewer Expertise:

NA

No decision status is available

# Reviewer report: Reviewer response for version 1

**DOI:** 10.21956/mep.19712.r29497

**Published:** 2018-11-10

**Authors:** Tan Nguyen

**Affiliations:** 1 Deakin University

This review has been migrated. The reviewer awarded 3 stars out of 5 This paper should be read for those interested in the area of IPE. Brief literature in the area was discussed, the methods used and the analysis of them was appropriate. However, I have reservations about the conclusions made because I felt that the evaluation only measured Level 1: Reaction education outcomes (Kirkpatrick model). The evaluation was also short term. It would be useful to understand the context of the IPE curriculum in terms how it aligns with the learning outcomes. i.e. what formative or summative assessments could have measured educational outcomes? The research should also discuss the natural effects of longitudinal learning as with most student cohorts, whether it has IPE or not, students skills will naturally improve regarding the soft skills and technical skills.

Reviewer Expertise:

NA

No decision status is available

# Reviewer report: Reviewer response for version 1

**DOI:** 10.21956/mep.19712.r29495

**Published:** 2018-11-10

**Authors:** Gominda Ponnamperuma

**Affiliations:** 1 Faculty of Medicine, University of Colombo

This review has been migrated. The reviewer awarded 3 stars out of 5 This is a paper on a very important initiative, and the paper is well written, in general. Please find below some feedback that may help you improve this paper.AbstractSome information on the statistical analysis should be included under ‘methods’.Please include the sample size and the response rate under results.The final sentence under ‘results’ gives the impression that there had been an intervention (i.e. a curriculum change/alteration) to improve IPE. If this is the case, then this is not merely a descriptive study but a pre and post interventional study. Then, the intervention should be described under ‘methods’. I see that in fact this is the case, as stated in the methods of the main article. Since this is the case, the authors need to decide how best to frame the study aim/objective (or the research question). One option is to report this as a pure descriptive study that evaluated the perceptions of dental and medical students. Another option would be to report this study as a pre-post intervention study. I see that the authors have decided on the first option. Hence, I would recommend either deleting the information on the curriculum change, or explicitly stating that evaluating the pre and post perception after modifying the curriculum also as a research aim.Main articleIntroduction: It would be good to give a reference to the sentence that states that IPE has been in existence since the 1970s.Methods: I would like some more information about the instrument (i.e. RIPLS) that was used to collect data.Please include the details of statistical analysis under ‘methods’.Results: In Table 4, can the results in year 1 and year 2 be presented side by side (in two adjacent or near by columns) along with the results of the application of an appropriate statistical test to verify whether these changes are significant?Discussion: Please compare the results of this study with those of similar studies. I am certain that there are many studies conducted using RIPLS alone.Conclusion: Please use this section only to answer the research aim(s)/question(s) and to highlight any recommendation. Please shift the other explanations (or explanation of the conclusions) to the ‘discussion’ section.Finally, please proof read the final version to correct any minor typographical errors; e.g. (1) ‘All analyses were performed….’ rather than ‘All analysis were performed….’(2) ‘The only RIPLS statement to which the medical student and dental students responded differently was….’ rather than ‘The only RIPLS question in which the medical student and dental students did not agree was….’(3) Omit redundant duplication of information such as ‘by Chi-square test, Fisher’s exact test, or Wilcoxon rank sum test. All analysis were performed in SAS 9.4 (Cary, NC)’. This is stated also within the ‘results’ section.(4) ‘This may be explained by the fact that older participants…..’ rather than ‘This may be explained by the fact that older patients…..’Please consider the above only as examples, rather than a comprehensive list of typographical omissions.

Reviewer Expertise:

NA

No decision status is available

# Reviewer report: Reviewer response for version 1

**DOI:** 10.21956/mep.19712.r29496

**Published:** 2018-11-08

**Authors:** Jayne Garner

**Affiliations:** 1 university of liverpool

This review has been migrated. The reviewer awarded 3 stars out of 5 I found many aspects of this paper useful and informative. As no expert on current IPE literature, the introduction provided useful recent background with regard to learning scales and identity formation. A wider selection of related literature could have provided clearer context for the paper more generally. The overall population size is small at 51, particularly for statistical testing. However, results are clearly presented and the constant comparison between the dental and medical student scores makes for interesting reading. Due to the small sample size it is hard to make wide generalisations about the IPE programme and its transferability. However, students found the learning useful and the constructive comment feedback is useful for anyone planning similar work in future.

Reviewer Expertise:

NA

No decision status is available
